# Supplementary material for: Enhanced identification of small molecules binding to hnRNPA1 via cryptic pockets mapping coupled with X-ray fragment screening
Source: J Biol Chem. 2025 Feb 19;301(4):108335. doi: 10.1016/j.jbc.2025.108335 (PMC11979464; doi:10.1016/j.jbc.2025.108335)
Supplement: Supporting Information [file mmc1.docx]

Table S1 – Summary of UP1 deposited structures. The table summarises all deposited UP1-fragment complexes structures.


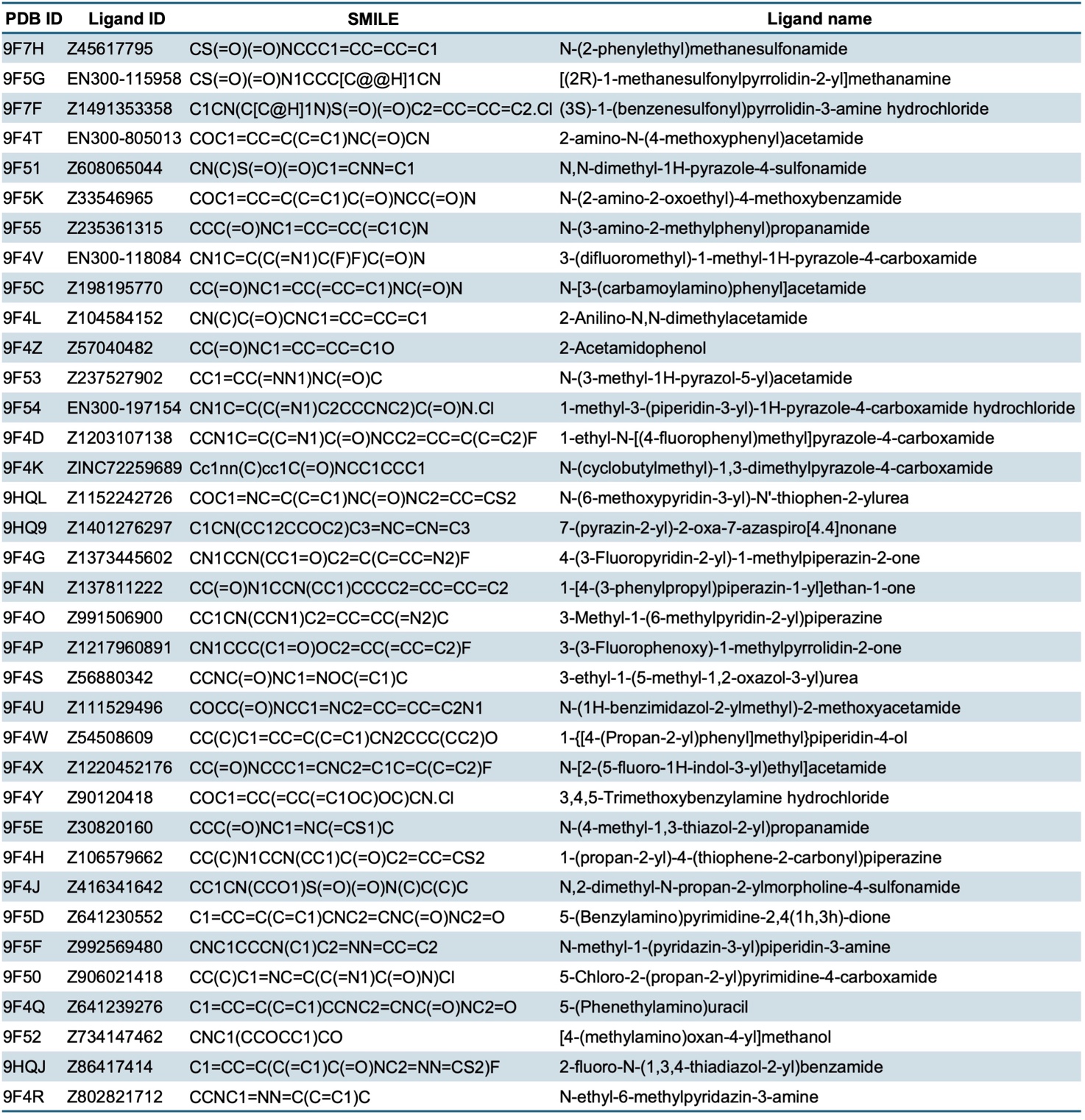


Table S2 – Crystallographic and refinement statistics – Statistics for the highest-resolution bin of reflections are in parentheses. Fragments Z106579662 and Z235361315 binds UP1 on two different surfaces, while two alternative conformations are visible for Z641230552


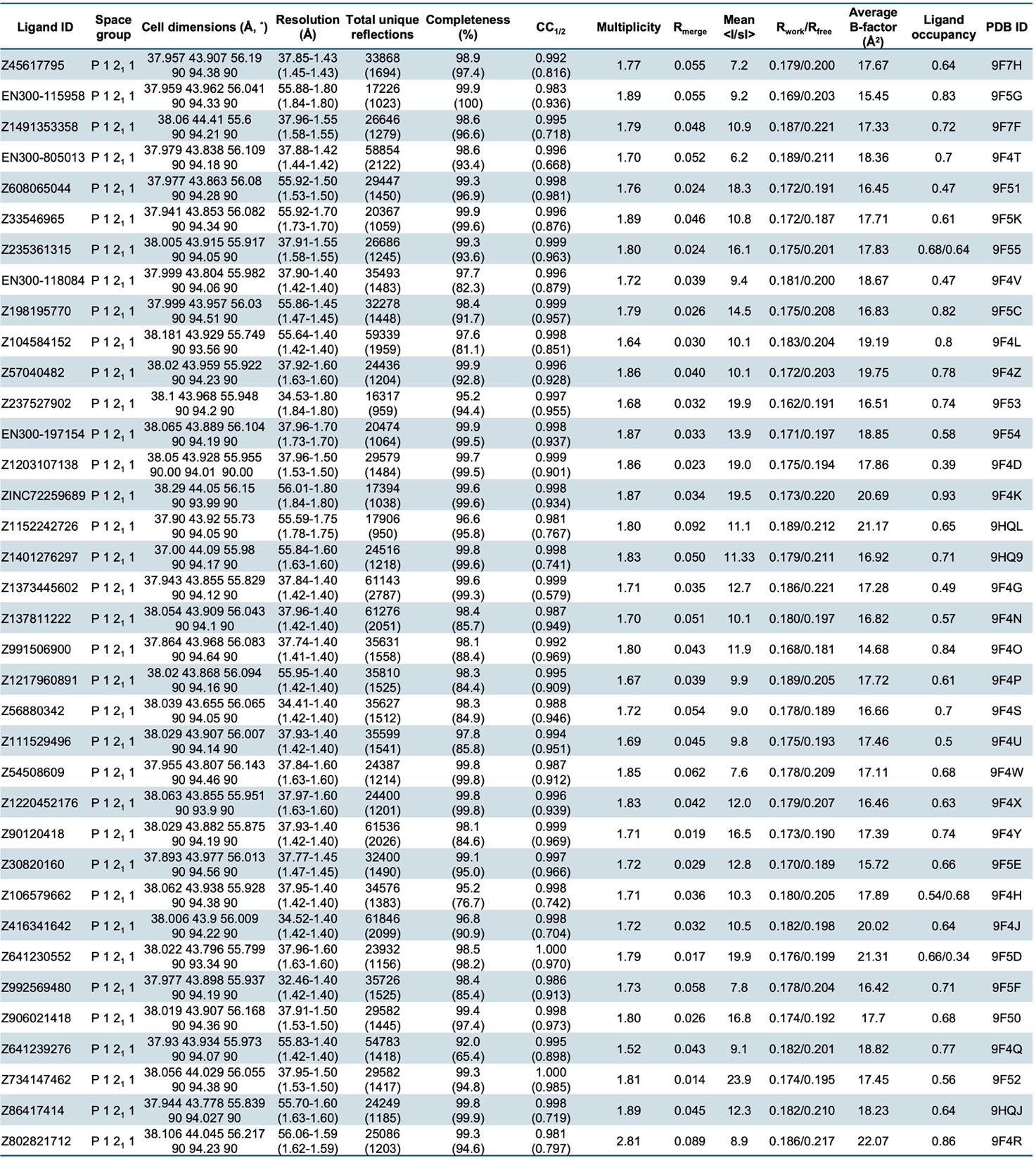


Table S3 – Identified fragments. The table summarises information regarding fragments identified to bind UP1, highlighting fragment induced changes in F17 and F59 side chains and inter-RRM loop conformations. Furthermore, fragment binding locations are indicated.


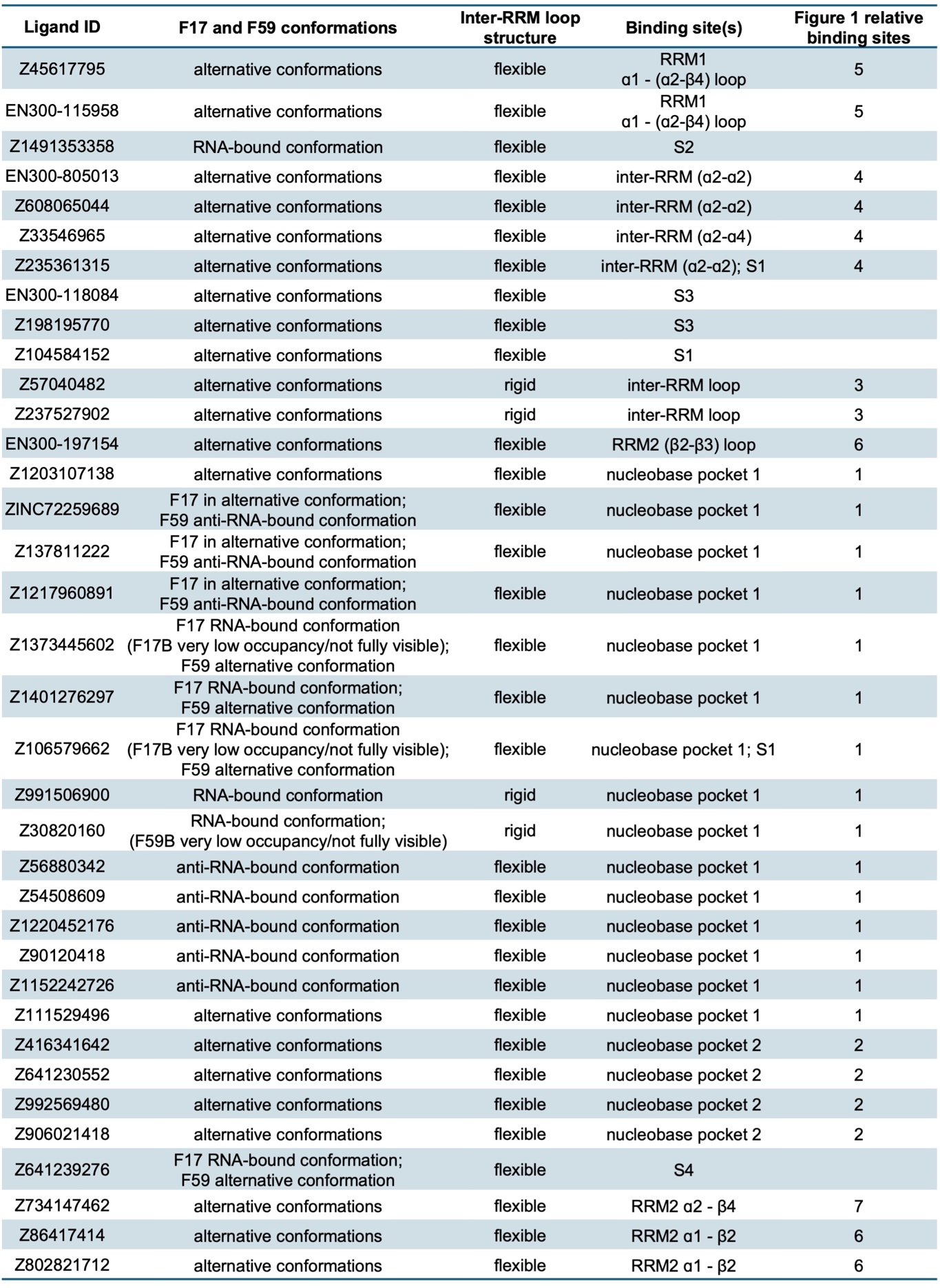


S1 – fragment sandwiched between RRM1 loop (β1-β3) and RRM2 ɑ1 of the symmetry related molecule; S2 – fragment sandwiched among 3 symmetry related molecules: inter-RRM interface, the RRM1 (β2 – β3) loop of one symmetry related molecules and the RRM2 (β2 – β3) loop of the second symmetry related molecule; S3 – fragment sandwiched between (interRRMs-loop – (ɑ5) loop) and (β5- β7) loop of the symmetry related molecule; S4 - fragment sandwiched between nucleobase pocket 2 and RRM2 α1 – β3 of the symmetry related molecule.

Table S4 – UP1-fragment interactions. UP1 residues forming contacts with fragments in the structures are listed by their position and by their single-letter identity. Interactions were identified using PyMol.


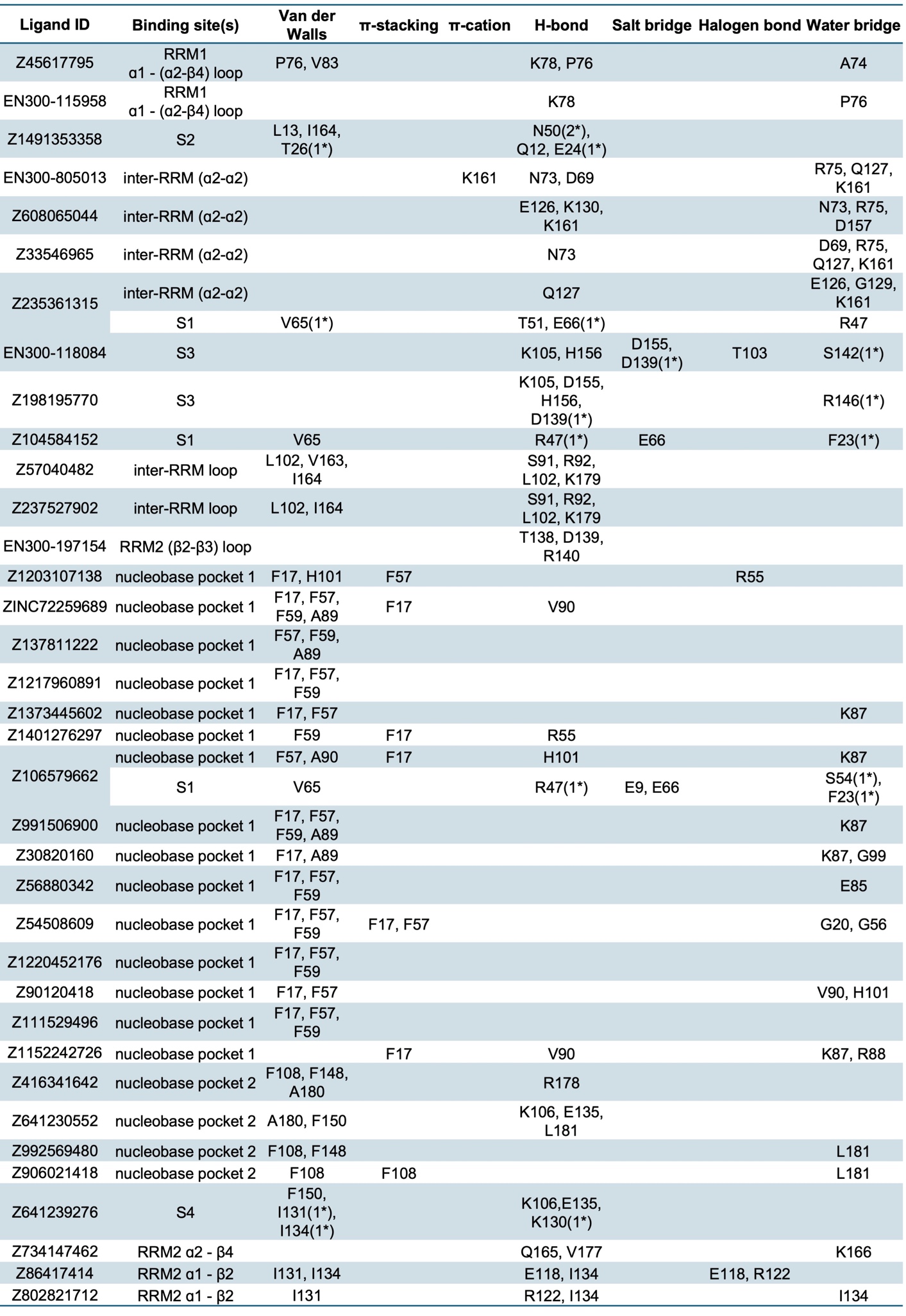


(n*) = symmetry related


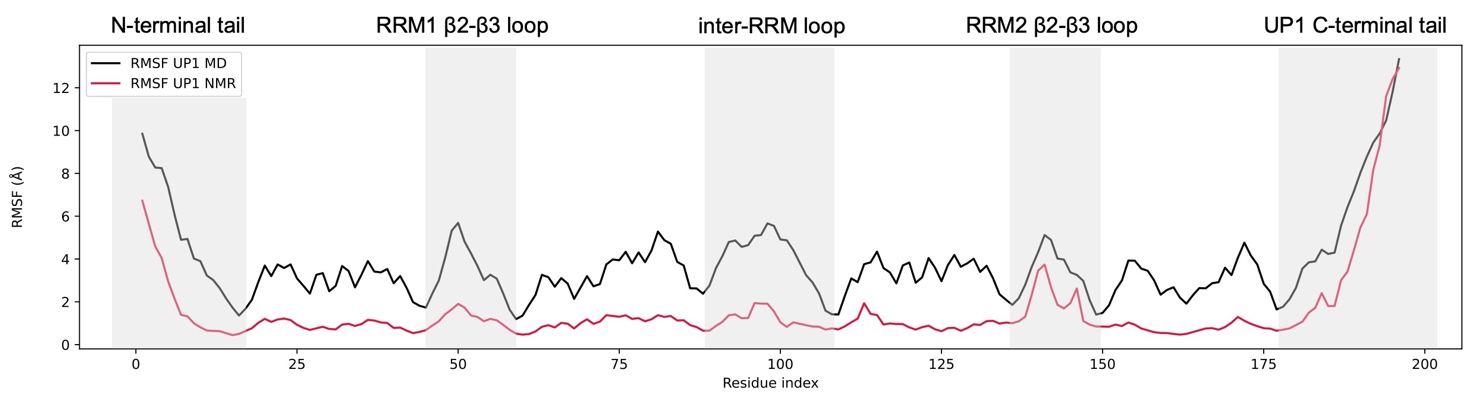


**Figure S1 Comparison of MD simulations and NMR data**. Per-residue root mean square fluctuation (RMSF) for the extended MD simulation (black line) was superimposed for direct comparison over the RMSF of the NMR ensemble (PDB ID: 2LYV) (red line). Flexible regions are highlighted with grey boxes.


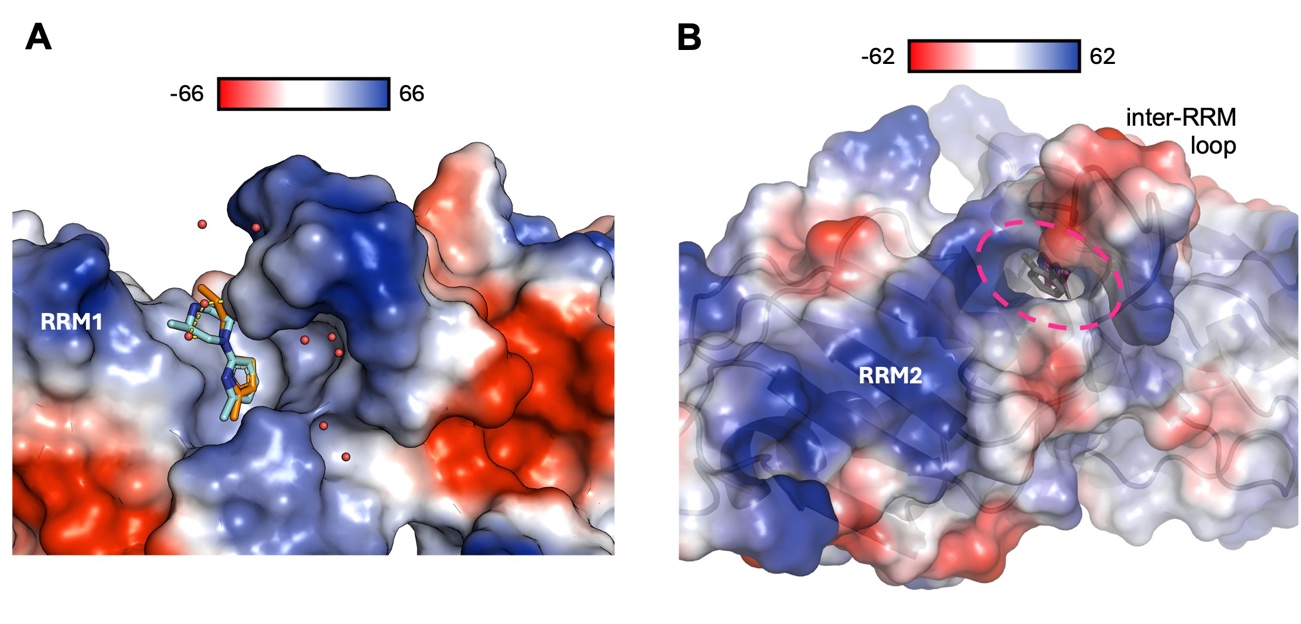


**Figure S2 Fragments induced conformational changes**. **A**) UP1 electrostatic surface showing the formation of a positive charged pocket at the edge on RRM1 upon binding of Z30820160 or Z991506900 to the nucleobase pocket 1. **B**) UP1 electrostatic surface showing the formation of a mostly neutral pocket (highlighted with a magenta dotted line) on the edge of RRM2 upon binding of Z57040482 and Z237527902 to the inter-RRM loop site.


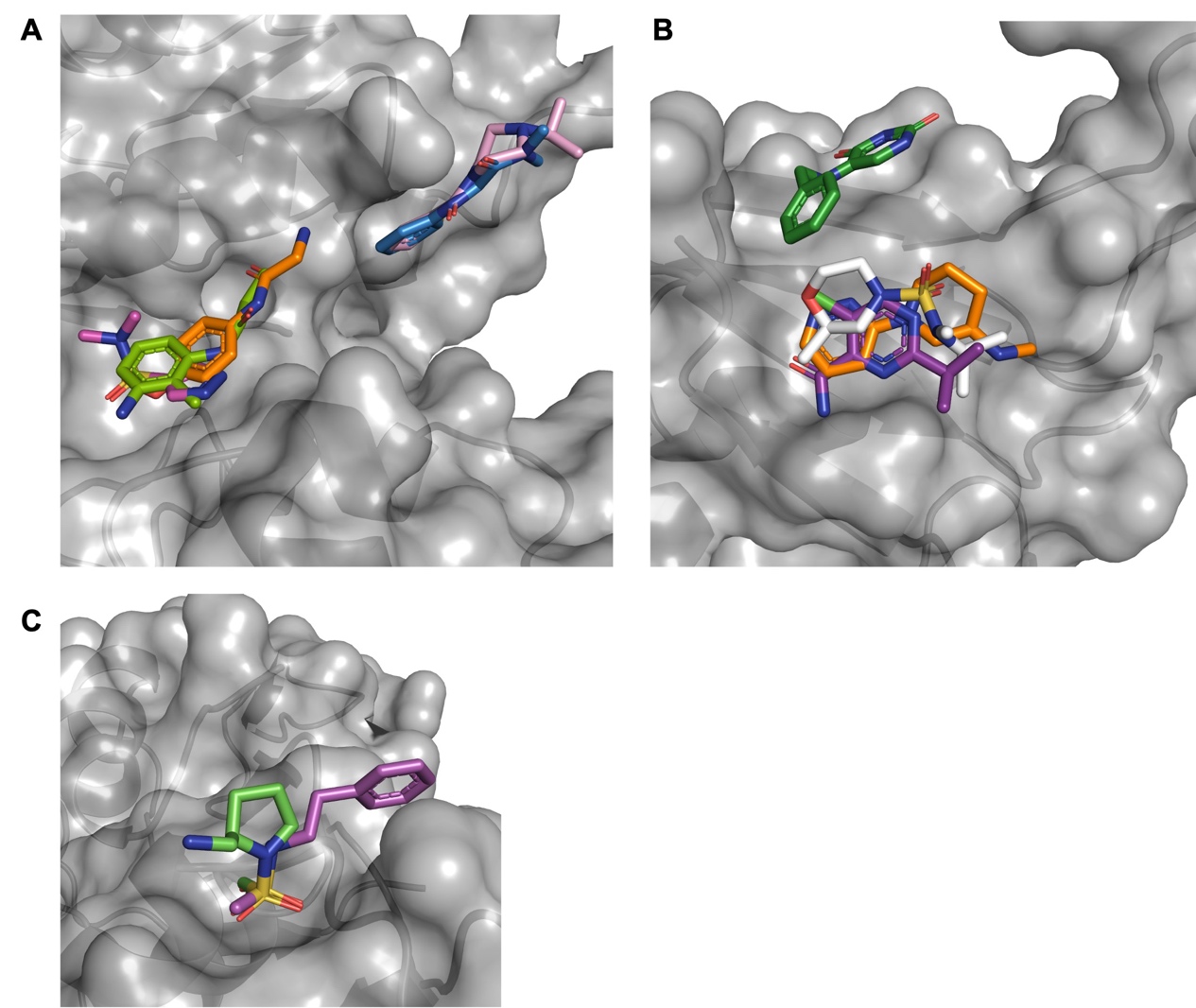


**Figure S3. Fragment merging and linking opportunities can be directly inferred for many hits. A**) Overlay of EN300-805013 (orange), Z608065044 (magenta), Z235361315 (green), Z104584152 (blue) and Z106579662 (pink). **B**) Z416341642 (white), Z992569480 (orange), Z906021418 (purple), Z641230552 (green). **C**) Overlay of Z45617795 (magenta) and EN300-115958 (green).


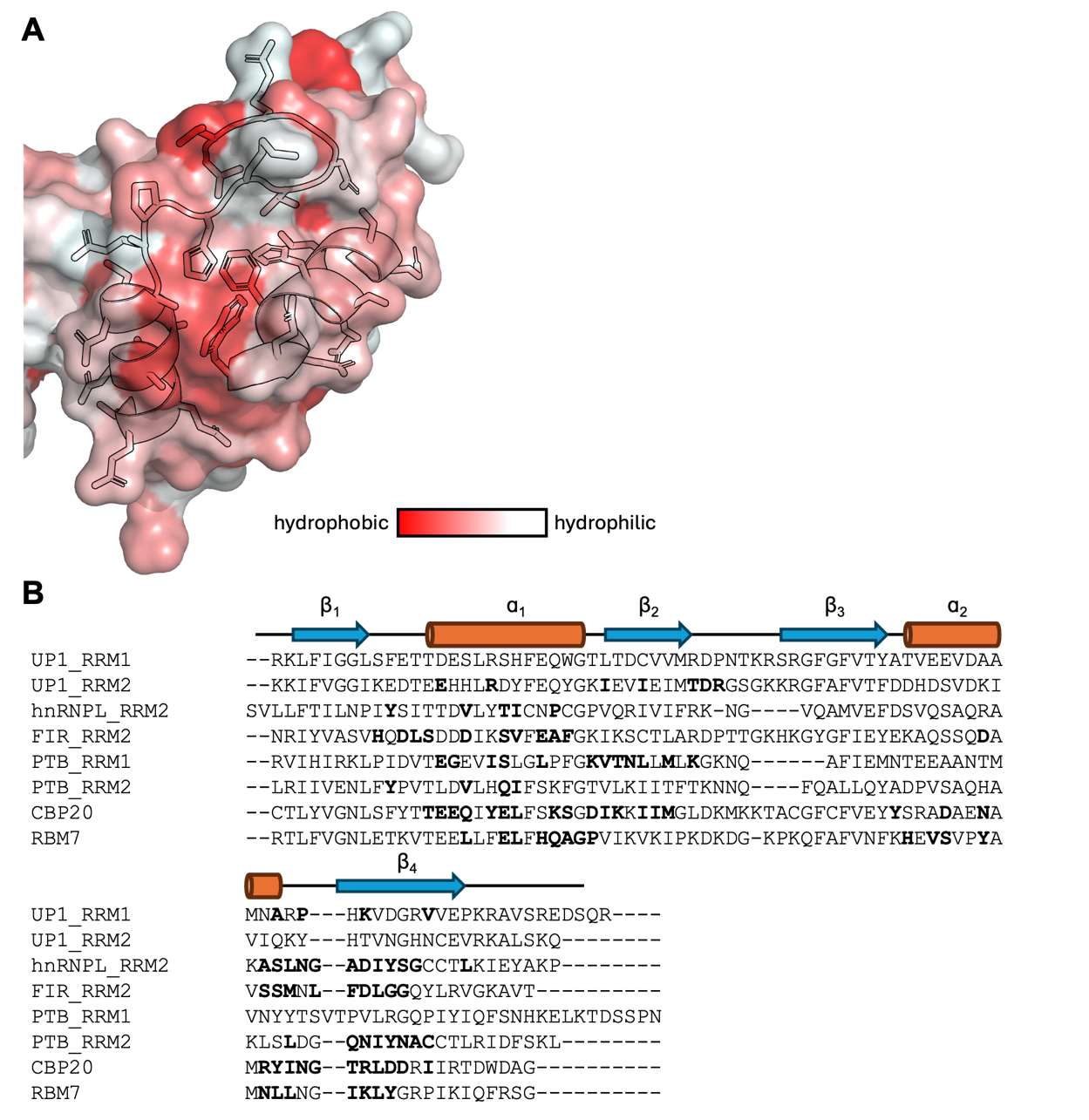


**Figure S4. RRM PPI surface. A**) Conserved hydrophobic depression on the predicted PPI surface defined by RRM1 α1 - α2 – (α2-β4) loop of UP1. UP1 RRM1 surface is coloured according to the Eisenberg hydrophobicity scale. **B**) Sequence alignment among the RRM1 and 2 of UP1 and the RRM domains of hnRNPL, FBP-interacting repressor (FIR), Polypyrimidine tract binding protein (PTB), Nuclear cap-binding protein subunit 2 (CBP20), RNA-binding protein 7 (RBM7) sequences, mapped against the conserved RRM secondary structure. In bold are highlighted residues mediating fragments-UP1 interactions in our structures and protein-protein/peptide interactions in hnRNPL, FIR, PTB RRM1 and RRM2, CBP20 and RBM7 structures (PDB ID 7EVR, 2KXH, 8BWF, 3ZZY, 5oo6, 5LXY, respectively).


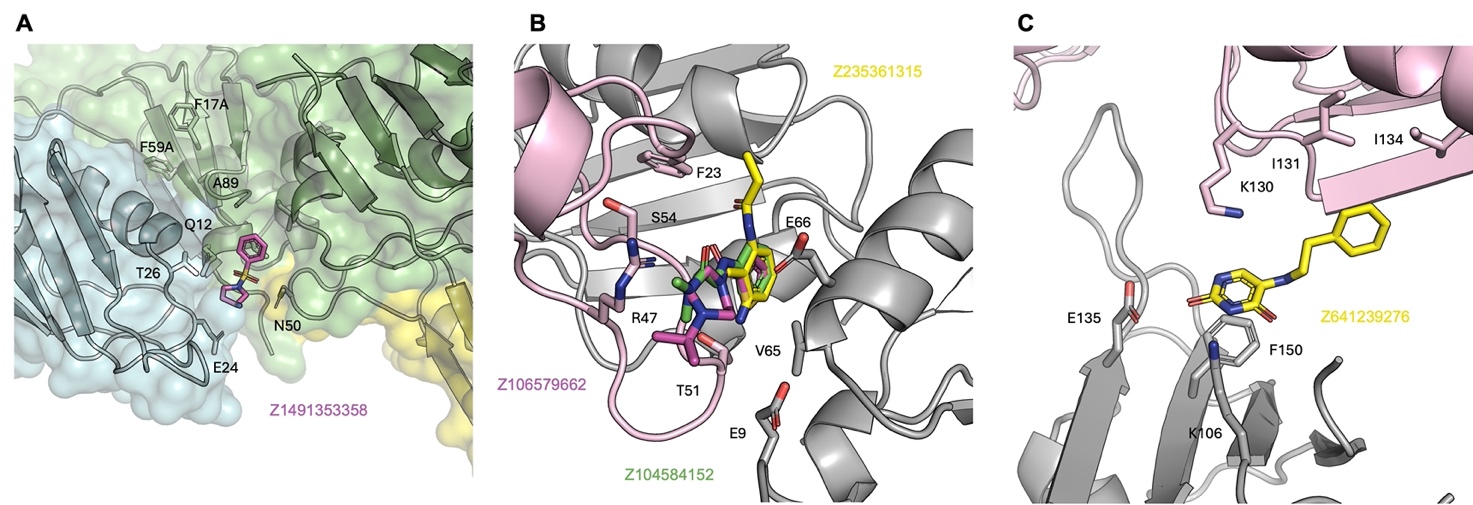


**Figure S5 Fragments Influenced by Crystal Contacts**. **A**) Z1491353358 (purple) bound on a pocket generated at the intersection of three symmetry-related molecules, and formed by the inter-RRM interface of molecules 1 (green), RRM1 (β2 – β3) loop of symmetry related molecules 1 (yellow) and RRM1 (β1 – ɑ1) loop of symmetry related molecule 2 (cyan). **B)** overlay of Z235361315 (yellow), Z104584152 (green), and Z106579662 (purple) bound to the inter-RRM interface (grey) and the RRM1 (β2 – β3) loop of the symmetry-related molecules (pink). **C**) Z641239276 (yellow) bound to the RRM2 nucleobase pocket (grey) and the RRM2 α1 – β3 of the symmetry-related molecules (pink).


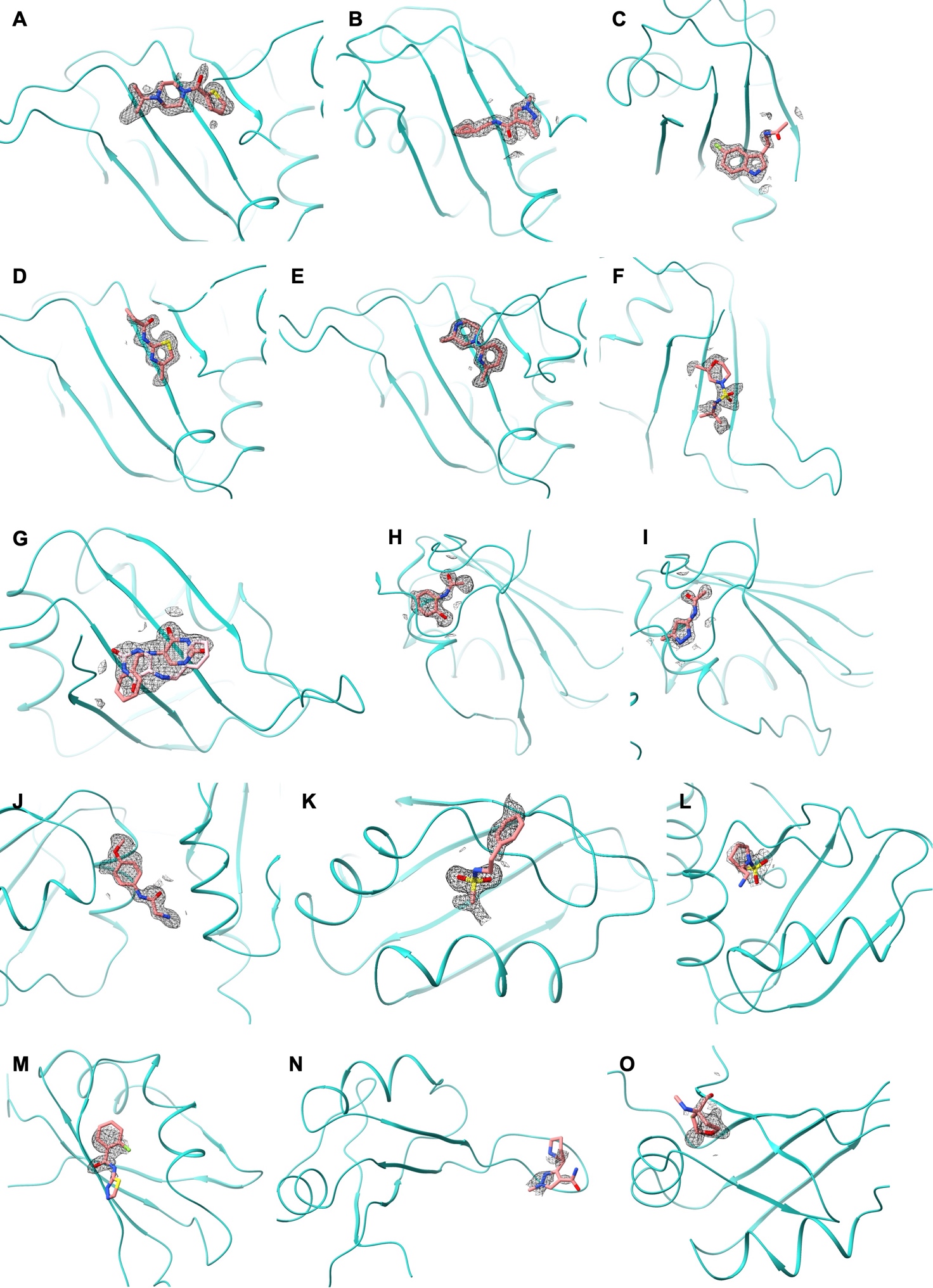


**Figure S6 Electron density maps of ligands in figure 2, 3 and 4**. The final electron density map calculated using mFo-DFc coefficients for **A**) Z106579662, **B**) ZINC72259689 **C**) Z1220452176, **D**) Z30820160, **E)** Z991506900, **F)** Z416341642, **G)** Z641230552, **H)** Z57040482, **I)** Z237527902, **J)** EN300-805013, **K)** Z45617795, **L)** EN300-115958, **M)** Z86417414, **N)** EN300-197154, **O)** Z734147462. Ligands are shown as sticks coloured by heteroatoms, with UP1 shown as cyan cartoon.
